# Habitat Suitability and Glacial Refugia of the Turbid-Water Coral *Turbinaria peltata* Across the Indo-West Pacific Ocean Based on MaxEnt Modeling

**DOI:** 10.3390/biology15181626

**Published:** 2026-09-15

**Authors:** Yanping Zhang, Chubin Shi, Jie Zhou, Li Liu, Quehui Tang, Jian Liao

**Affiliations:** 1 College of Fisheries, Guangdong Ocean University, Zhanjiang 524088, China; ypzhang@gdou.edu.cn (Y.Z.); scb120@stu.gdou.edu.cn (C.S.); zhoujielyg@sina.com (J.Z.); zjouliuli@163.com (L.L.); 2 Guangdong Laboratory of Southern Ocean Science and Engineering, Zhanjiang 524088, China; 3 Key Laboratory of Aquaculture in the South China Sea for Aquatic Economic Animal of Guangdong Higher Education Institutes, College of Fishery, Guangdong Ocean University, Zhanjiang 524088, China; 4 South China Sea Fisheries Research Institute, Chinese Academy of Fishery Sciences, Guangzhou 510300, China; tangquehui@scsfri.ac.cn

**Keywords:** species distribution modeling, last glacial maximum, scleractinian coral, benthic ecology, marine conservation, paleobiogeography

## Abstract

As a typical turbid-water reef coral, *Turbinaria peltata* shows strong tolerance to high temperature and sediment disturbance, yet its basin-scale niche drivers and glacial distribution dynamics across the Indo-West Pacific remain unclear. Here we constructed optimized MaxEnt models using 322 independent distribution points and 21 non-collinear marine environmental variables for modern and Last Glacial Maximum (LGM) scenarios. The model average test AUC reached 0.9874, showing excellent predictive performance. Water depth acted as the most influential predictor in the model (contribution = 44.76%), followed by distance to land and warm-season sea temperature; habitat suitability had an extremely weak linear correlation with depth (*R*^2^ = 0.05), consistent with a strongly nonlinear response along the depth gradient. Modern suitable habitats are mainly concentrated in Southeast Asia and northern Australia, with the South China Sea at the species’ northern margin. Massive shelf exposure during the LGM severely shrank its range, and three potential glacial refugia (northeastern Australia, Madagascar coast, Red Sea–Gulf of Aden) were identified. These refugia may have maintained coral populations through glaciation and contributed to the present-day biogeographic patterns of this species. Our findings provide quantitative spatial evidence for targeted conservation of nearshore stress-resistant corals under climate warming.

## Simple Summary

Turbid-water coral *Turbinaria peltata* has strong stress resistance, yet its basin-scale distribution rules and glacial history are unclear. We constructed reliable MaxEnt models (test AUC = 0.9874) for modern and LGM scenarios. Water depth dominates its distribution, and its core modern habitats lie in Southeast Asia and northern Australia. Severe habitat contraction occurred in the LGM, with three candidate glacial refugia identified. Our results provide spatial evidence for targeted coral conservation amid climate warming.

## 1. Introduction

Coral reefs constitute the most biodiverse marine ecosystems on Earth and provide irreplaceable ecological services including fishery resources, coastal wave attenuation and carbon sequestration [1]. Nevertheless, global climate warming, ocean acidification, coastal eutrophication and human coastal exploitation have triggered widespread coral bleaching, population recession and habitat fragmentation over recent decades [2,3]. Most scleractinian coral species with high light and clear-water requirements suffer severe population declines, while stress-tolerant turbid-water corals of the genus *Turbinaria* display stronger resistance to thermal disturbance and sediment loading, emerging as critical indicator taxa for reef ecosystem resilience under anthropogenic and climatic pressures [4,5]. Among this genus, *Turbinaria peltata* is a widespread reef-building coral endemic to the Indo-West Pacific tropical marine realm, capable of surviving across a broad bathymetric gradient and turbid nearshore environments that are unsuitable for most sensitive reef corals. Its unique physiological tolerance makes it an ideal model to disentangle how marine environmental factors shape the biogeography of resilient coral species, as well as to reconstruct historical range shifts during paleoclimatic fluctuations such as the Last Glacial Maximum (LGM) [6].

To date, existing research on *T. peltata* has predominantly focused on laboratory physiological experiments, local community surveys, and population genetic analysis [7]. Few studies have systematically quantified the relative importance of multi-dimensional marine physical, chemical, and nutrient variables regulating its large-scale spatial distribution across the entire Indo-West Pacific basin. Moreover, the core environmental drivers determining its habitat suitability remain unclear, especially the bathymetric constraint widely observed in field surveys but lacking quantitative modeling verification. In addition, paleoclimatic simulations of reef coral refugia mainly target clear-water Acropora-dominated reefs [8]. The glacial distribution dynamics and long-term stable refugia of turbid-water stress-resistant corals like *T. peltata* are still poorly documented, limiting our understanding of the evolutionary biogeography of nearshore coral species. Species distribution models (SDMs), particularly the maximum entropy algorithm (MaxEnt), serve as robust tools to map potential suitable habitats of marine organisms, quantify environmental variable contributions, and reconstruct paleodistributions under historical climate backgrounds [9,10], offering an effective solution to fill the above research gaps.

In this study, we integrated global georeferenced occurrence records of *T. peltata* and high-resolution global marine environmental datasets [11,12] to construct MaxEnt distribution models covering two key time periods: contemporary marine conditions and the LGM. We first assessed the predictive performance of the optimized MaxEnt model, quantified the relative contribution and permutation importance of 21 non-collinear marine environmental predictors, and further analyzed the correlation between the dominant factor (water depth) and habitat suitability. Next, we simulated the global spatial pattern of current suitable habitats of *T. peltata* and clarified its biogeographic distribution characteristics across the Indo-West Pacific. Finally, we reconstructed the range contraction and fragmentation of potential habitats during the LGM, and identified three long-term candidate glacial refugia that sustained persistent coral populations through glacial cooling and sea-level fall. This work aims to (1) reveal the key marine environmental filters governing the distribution of the turbid-water coral *T. peltata*; (2) illustrate its contemporary suitable habitat zoning across the Indo-West Pacific; (3) characterize its historical range shifts and delineate critical glacial refugia during the last glaciation. Collectively, our findings provide quantitative theoretical evidence for the biogeographic formation mechanism of nearshore stress-tolerant corals and deliver targeted spatial references for the regional conservation and management of *T. peltata* coral reef resources under ongoing climate change.

## 2. Materials and Methods

### 2.1. Collection and Preprocessing of Distribution Data of T. peltata

**Figure 1:**
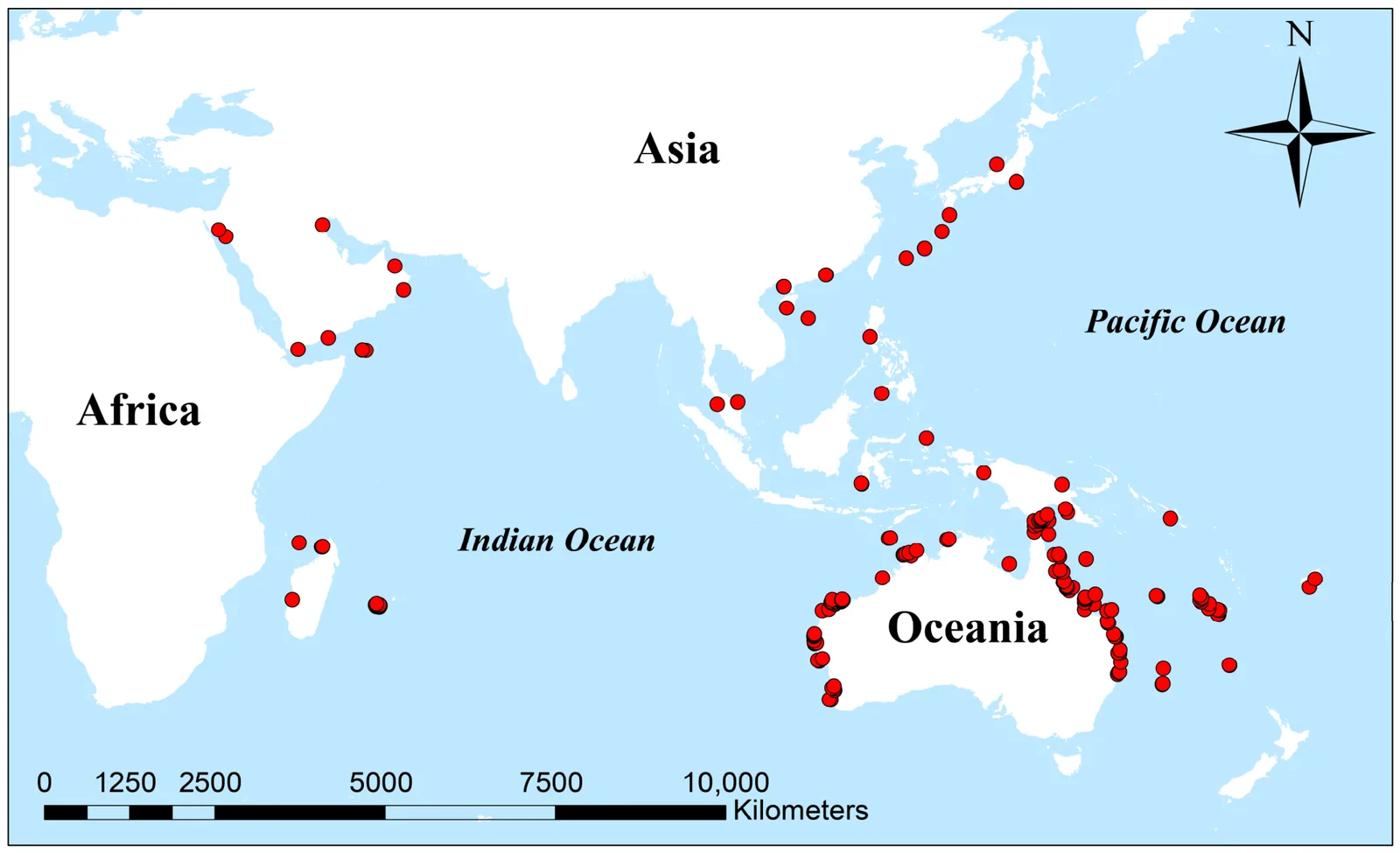
Distribution Maps of *T. peltata*. Red dots denote the final occurrence points used for MaxEnt modeling; white and light-blue areas indicate landmasses and oceans, respectively, within the Indo-West Pacific study extent (Africa, Asia, Oceania, Indian Ocean and Pacific Ocean).

The occurrence coordinate data of *T. peltata* were mainly retrieved from two authoritative global marine biodiversity databases, namely the Global Biodiversity Information Facility (GBIF, https://www.gbif.org/) and the Ocean Biodiversity Information System (OBIS, https://obis.org/). Supplementary valid distribution records were further supplemented by systematic collation of published peer-reviewed studies, field survey datasets from coral reef investigations across the Indo-Pacific seas conducted during 2021–2025, and museum specimen archive records. To unify data formats and facilitate subsequent spatial matching with environmental raster layers, all collected geographic coordinates recorded in sexagesimal (degree-minute-second) format were converted into decimal latitude and longitude format with ArcGIS 10.8 software (ESRI, Redlands, CA, USA) following standardized spatial coordinate calibration procedures [13]. During the data cleaning procedure, each occurrence record was verified one by one against the confirmed natural geographic range of *T. peltata* across the Indo-Pacific Ocean. Duplicate coordinate points, geographically anomalous records located on land or outside verified marine distribution zones, and records with missing or obviously biased latitude and longitude information were eliminated strictly [14]. Taxonomic identification of each record was cross-checked against the original source information and, where necessary, the World Register of Marine Species (WoRMS); records with uncertain or unsupported taxonomic identification were excluded. To mitigate spatial autocorrelation and spatial aggregation bias, which may induce model overfitting in subsequent MaxEnt modeling, a grid-based spatial thinning strategy was implemented: the entire Indo-Pacific study area was divided into 5 km × 5 km regular grid cells. Only one occurrence point closest to the geometric center of each grid cell was retained, which effectively reduced spatial clustering of sampling points while maintaining the overall spatial distribution pattern of this coral species [15]. After multi-step screening, spatial verification, and thinning operations, the final valid dataset of *T. peltata* consisted of 322 geographically independent occurrence coordinates (see Table S1 for detailed coordinate information). The cleaned distribution dataset was imported into ArcGIS 10.8 for spatial visualization and mapping, which illustrated the basin-wide geographic extent of the occurrence records, the land–ocean configuration of the study area, and the spatial clustering pattern across the Indo-West Pacific (Figure 1).

### 2.2. Oceanic Environmental Data Collection and Processing

Oceanic environmental variables are crucial for defining species niches. We downloaded oceanic environmental variables from the Global Marine Environment Datasets (GMED, http://gmed.auckland.ac.nz/, accessed on 24 April 2022) (detailed variables shown in Table 1), with a resolution of 2.5 arc-minutes (approximately 4.6 km at the equator), and all layers were resampled to this common resolution before analysis; the 5 km × 5 km thinning cell size was chosen to approximate the raster resolution while removing redundant occurrences within the same environmental cell. Such high-resolution global marine environmental layers have been widely applied in marine niche modeling, including MARSPEC [11] and Bio-ORACLE [12]. Using the “Extract Multi Values to Points” spatial analysis tool in the ArcGIS toolbox, we extracted the values of these oceanic environmental variables at each occurrence point of *T. peltata*. In R, we performed variance inflation factor analysis using the *usdm* 1.1–18 package to iteratively eliminate multicollinearity among predictors; variables with a VIF ≥ 10 were removed one by one, starting from the predictor with the highest VIF value, until all retained variables satisfied VIF < 10 [16]. A total of 21 non-collinear environmental variables were ultimately retained and utilized for subsequent MaxEnt modeling analysis of *T. peltata* under the Last Glacial Maximum and current conditions. For the LGM scenario, palaeoenvironmental layers of the same 21 predictors were reconstructed from palaeoceanographic simulations representative of approximately 21,000 years before present (Marine Isotope Stage 2) and were processed following the identical pipeline (geographic extent, spatial resolution and layer alignment) as the contemporary layers. To account for the eustatic sea-level lowering of 120–135 m during the LGM, the palaeo-land–sea configuration was reconstructed by subtracting the glacial sea-level fall from the present-day bathymetric grid, and the LGM layers were masked to this recon-structed coastline before modeling.

### 2.3. Model Construction and Evaluation

**Figure 2:**
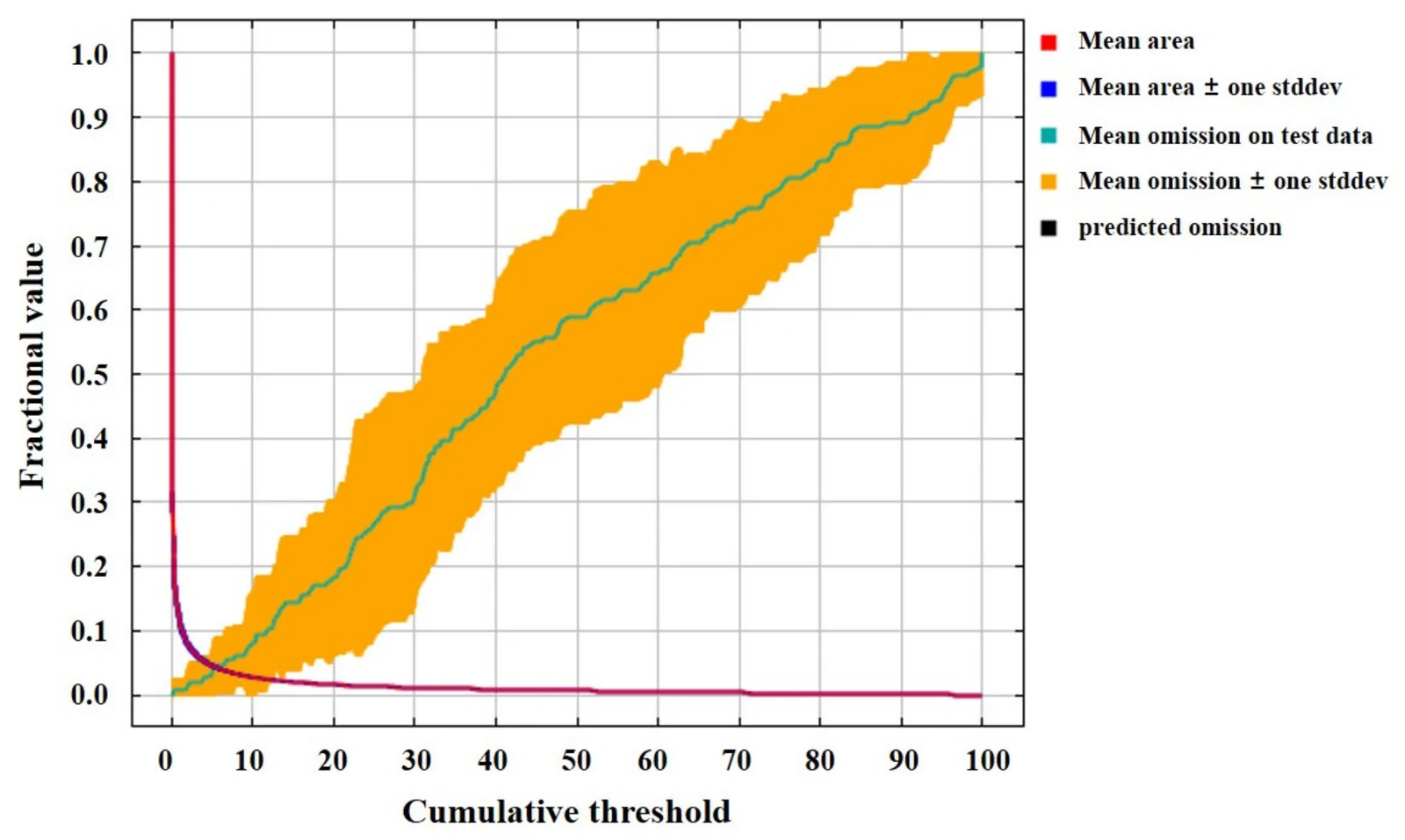
Average omission and predicted area for *T. peltata*. The x-axis represents the cumulative suitability threshold, and the y-axis denotes the fractional value. Red line: mean area predicted as suitable habitat; blue line: mean area ± one standard deviation; cyan line: mean omission calculated on test data; orange shaded region: mean omission ± one standard deviation across replicate model runs; black line: predicted theoretical omission.

This research adopted the maximum entropy algorithm (MaxEnt) to couple georeferenced occurrence records and screened marine environmental predictors. MaxEnt 3.4.1 [10] was applied to simulate the potential suitable habitats of *T. peltata* across two temporal phases: the Last Glacial Maximum and contemporary climate conditions. The model calibrated with the contemporary occurrence data was then projected onto the LGM environmental layers; because such a transfer may involve extrapolation into environmental combinations not represented in the calibration space, the LGM projections should be interpreted with appropriate caution. For model training and verification, 75% of valid occurrence records were randomly assigned as the training dataset, and the leftover 25% served as held-out test data; this partitioning procedure was repeated 10 times to improve model robustness [17]. Although random partitioning cannot fully eliminate spatial autocorrelation between the training and test sets across such a broad study area, the 5 km × 5 km spatial thinning applied before partitioning reduces the chance that records sharing nearly identical environmental conditions are split between the two sets; we acknowledge that a spatially explicit (e.g., spatial block) cross-validation would provide a more conservative evaluation and is recommended in future work. The iteration of the MaxEnt program stopped once the convergence threshold of 0.00001 was satisfied. Model complexity was controlled by the default auto-feature mode of MaxEnt 3.4.1, in which the feature classes (linear, quadratic, product, threshold and hinge) were automatically selected according to the training sample size, with the default regularization multiplier of 1.0 retained to prevent overfitting [10]. Model predictive performance was assessed via threshold-independent receiver operating characteristic (ROC) curves. The area under the ROC curve (AUC) acted as the core metric for judging model reliability, with evaluation criteria set as follows: excellent predictive capacity (AUC > 0.9), good performance (0.8 ≤ AUC < 0.9), moderate performance (0.7 ≤ AUC < 0.8), poor performance (0.6 ≤ AUC < 0.7), and invalid simulation (AUC < 0.6) [17]. A jack-knife resampling test was further implemented to quantify the relative contribution, permutation importance, and unique predictive information (as measured by the training gain, test gain, and AUC) of each environmental predictor driving the species distribution. In addition to the test AUC, model reliability was further corroborated by the test omission rate and the fraction of predicted area across cumulative suitability thresholds (Figure 2), which together provided complementary evaluation metrics beyond the test AUC. Clamping was set to the default value and the logistic output format was adopted. For each replicate, 10,133 background points were randomly sampled across the entire Indo-West Pacific study extent without restriction to the species’ known range, following the default background procedure of MaxEnt [10]; the multi-source occurrence data were spatially thinned at 5 km × 5 km to mitigate sampling bias.

Raw simulation outputs in ASC raster format were imported into ArcGIS 10.8 for subsequent spatial processing and suitability zoning. According to continuous habitat suitability indices generated by the model, the study area was categorized into four grades: highly suitable regions (suitability > 0.6), moderately suitable regions (0.4 < suitability ≤ 0.6), low-suitability marginal habitats (0.2 < suitability ≤ 0.4), and entirely unsuitable zones (suitability ≤ 0.2) [13]. Candidate glacial refugia for *T. peltata* were delineated as regions with predicted suitability values higher than 0.8 during the Last Glacial Maximum [17]. Spatial statistics tools embedded in the ArcGIS toolbox were used to calculate the occupied area of each suitability grade in the three periods, which offers theoretical support for targeted conservation management of this nearshore stress-resistant scleractinian coral.

## 3. Results

### 3.1. Model Performance

The MaxEnt model demonstrated excellent predictive performance for simulating the potential suitable habitats of *T. peltata* across the Indo-Pacific Ocean. Based on 241 training occurrence records and 81 held-out test samples randomly partitioned from the 322 spatially thinned occurrence records (75%/25%), against 10,133 background points, the average training AUC value reached 0.9908 ± 0.0002, and the average test AUC value attained 0.9874 ± 0.0049 across ten replicate runs. Both training and test AUC values exceeded 0.98, falling into the “excellent” performance category, indicating that the model possessed extremely high discrimination capacity in separating the recorded occurrences from background samples. Nevertheless, because MaxEnt is a presence-only method, AUC quantifies discrimination against background pseudo-absences rather than true absences, and a high AUC should not be interpreted as direct evidence of ecological accuracy of the predicted distribution. The low standard deviation of AUC values further confirmed the high stability and reproducibility of the model outputs. The model converged after an average of 1670 iterations, ensuring sufficient algorithmic optimization.

The average omission-predicted area plot further supported reliable model behavior (Figure 2). The observed omission calculated from test data increased gradually with rising cumulative suitability threshold, accompanied by a sharp reduction in the fraction of predicted suitable area. The observed test-data omission closely followed the expected theoretical omission trend, with moderate variability among replicates reflected by the orange ± one-standard-deviation envelope, and no strong sign of overfitting was detected. Overall, the MaxEnt model performed robustly and reliably in this study, providing a solid methodological foundation for subsequent habitat suitability analysis and environmental factor interpretation.

### 3.2. Key Environmental Factors Influencing the Potential Habitats of T. peltata

**Figure 3:**
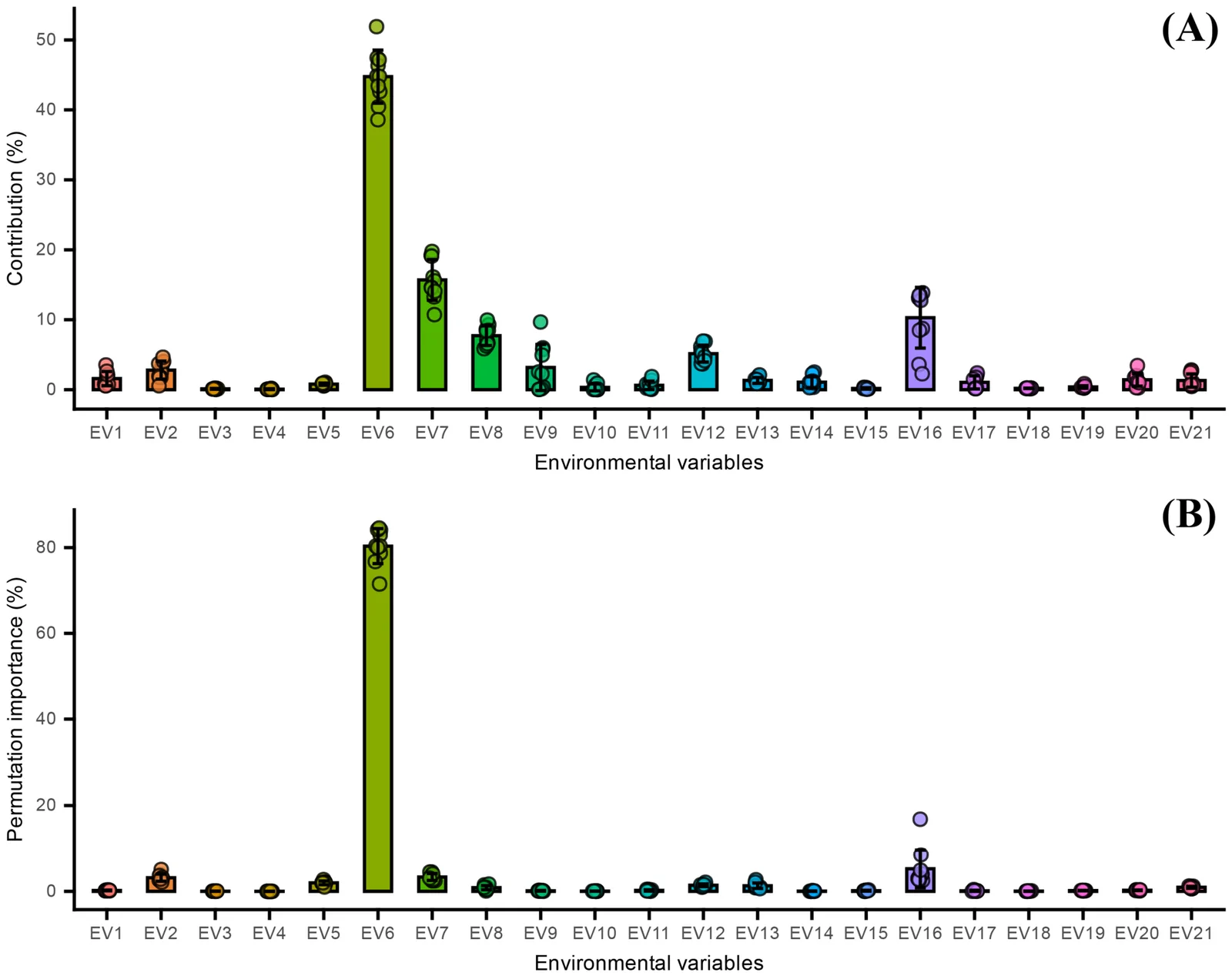
Contribution percentage (**A**) and permutation importance (**B**) of 21 marine environmental variables for habitat suitability of *T. peltata*. EV1, bottom dissolved oxygen; EV2, bottom silicate; EV3, minimum chlorophyll-a; EV4, chlorophyll-a range; EV5, maximum chlorophyll-a; EV6, depth; EV7, distance to land; EV8, nitrate; EV9, dissolved oxygen; EV10, saturated oxygen; EV11, photosynthetically active radiation; EV12, pH; EV13, primary productivity; EV14, mean salinity; EV15, silicate; EV16, sea surface temperature (May–Oct); EV17, maximum temperature; EV18, surface current; EV19, average tide; EV20, wave height; EV21, wind speed.

Highly collinear predictors were progressively excluded according to the VIF threshold criterion, and 21 non-collinear marine environmental variables were ultimately retained and incorporated into the MaxEnt model for habitat suitability simulation (Figure 3). Among these retained variables, depth (water depth) emerged as the most dominant factor shaping the distribution pattern of *T. peltata*, contributing 44.76% to the model and accounting for 80.34% of the permutation importance. This overwhelmingly high contribution indicated that water depth was the most influential predictor in the fitted model, although variable contribution and permutation importance describe the importance of a predictor within the model and do not by themselves demonstrate a causal ecological relationship; this pattern nevertheless reflects the strong dependence of this scleractinian coral on specific depth ranges for light availability, substrate stability, and hydrodynamic conditions. Land distance ranked as the second most important variable, with a contribution rate of 15.70% and a permutation importance of 3.32%, highlighting the nearshore coastal preference of *T. peltata* and its adaptation to turbid, terrigenous-influenced coastal waters. Sea surface temperature from May to October (sst_mayoct) was the third critical contributing factor (10.29% contribution, 5.25% permutation importance), underscoring that warm-season thermal conditions played a crucial role in regulating the survival, growth, and geographic distribution of this tropical coral species. Nitrate concentration (7.72%) and pH (5.15%) also exerted notable influences on habitat suitability, reflecting the importance of nutrient status and seawater carbonate chemistry for coral physiology. The remaining environmental variables, including dissolved oxygen, silicate, wave height, primary productivity, and salinity, made relatively minor contributions (each < 5%). The jack-knife test results further corroborated that depth contained the most unique information for predicting *T. peltata* distribution, while variables such as chlorophyll-a and surface current contributed the least unique information. Collectively, the distribution of *T. peltata* was primarily driven by bathymetric features, coastal proximity, and warm-season thermal regimes, consistent with the ecological characteristics of this stress-tolerant, nearshore turbid-water coral species.

**Figure 4:**
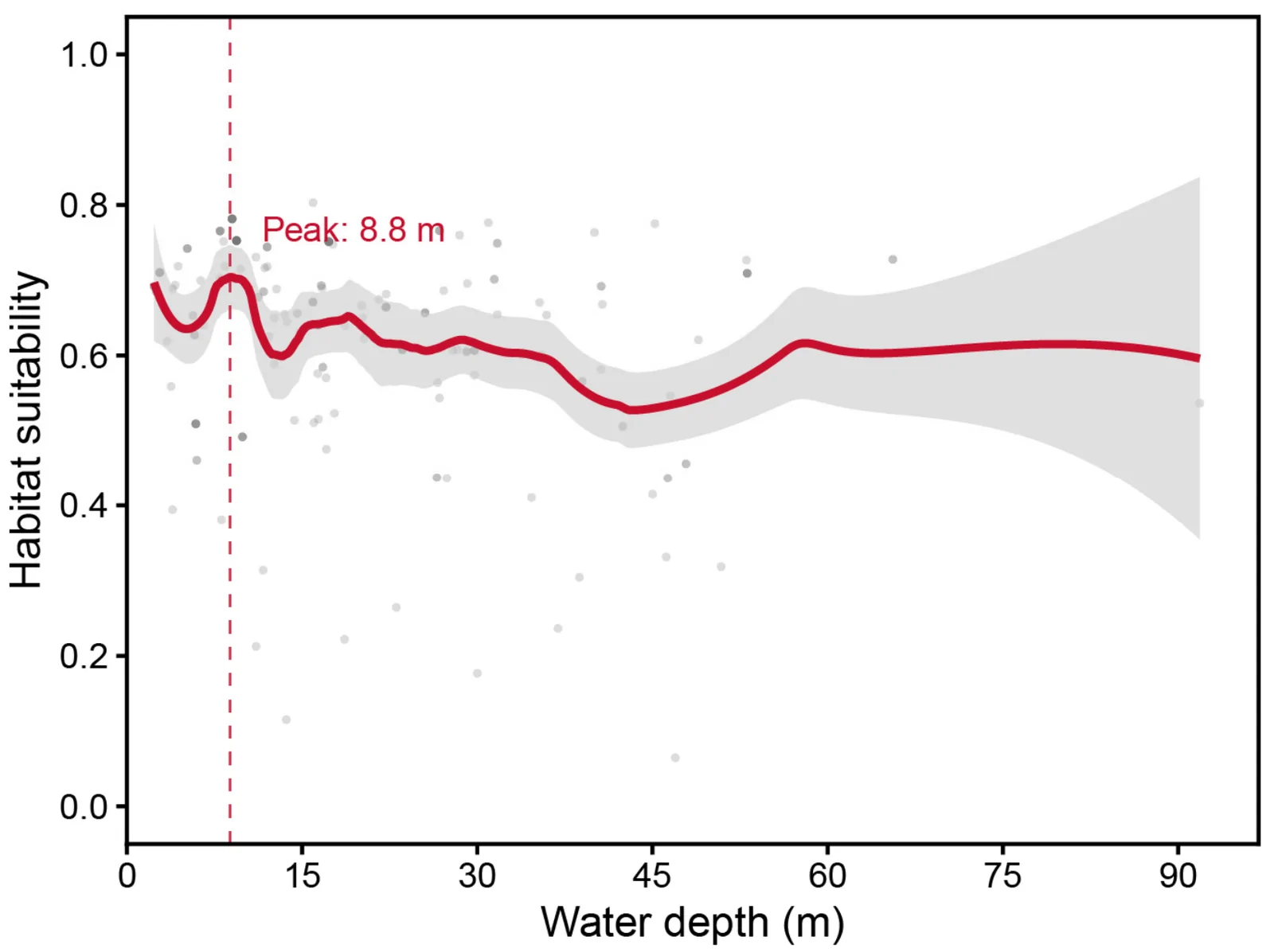
Response curve of habitat suitability of *T. peltata* along the water-depth gradient (EV6, depth). Gray dots represent the raw model output points; the red solid line is the LOESS smoothing fit (span = 0.4); the gray shaded band indicates the 95% confidence interval of the fit; and the red dashed line with label marks the peak suitability (0.704) at the optimal water depth of 8.8 m.

Habitat suitability of *T. peltata* varied markedly along the water-depth gradient (Figure 4). Suitability was highest in shallow waters, reaching a peak of approximately 0.70 at 8.8 m and remaining high within the upper 20 m (mean 0.655), where the raw maximum suitability (0.803) also occurred at ~16 m. Beyond this optimum, suitability declined progressively with increasing depth and bottomed out at approximately 0.53–0.55 around 45–50 m, before recovering gradually and leveling off at about 0.60 towards the deepest records (~90 m). Overall, the response curve reveals a clear preference of *T. peltata* for shallow, nearshore waters.

### 3.3. Simulation of Current Suitable Habitats of T. peltata

**Figure 5:**
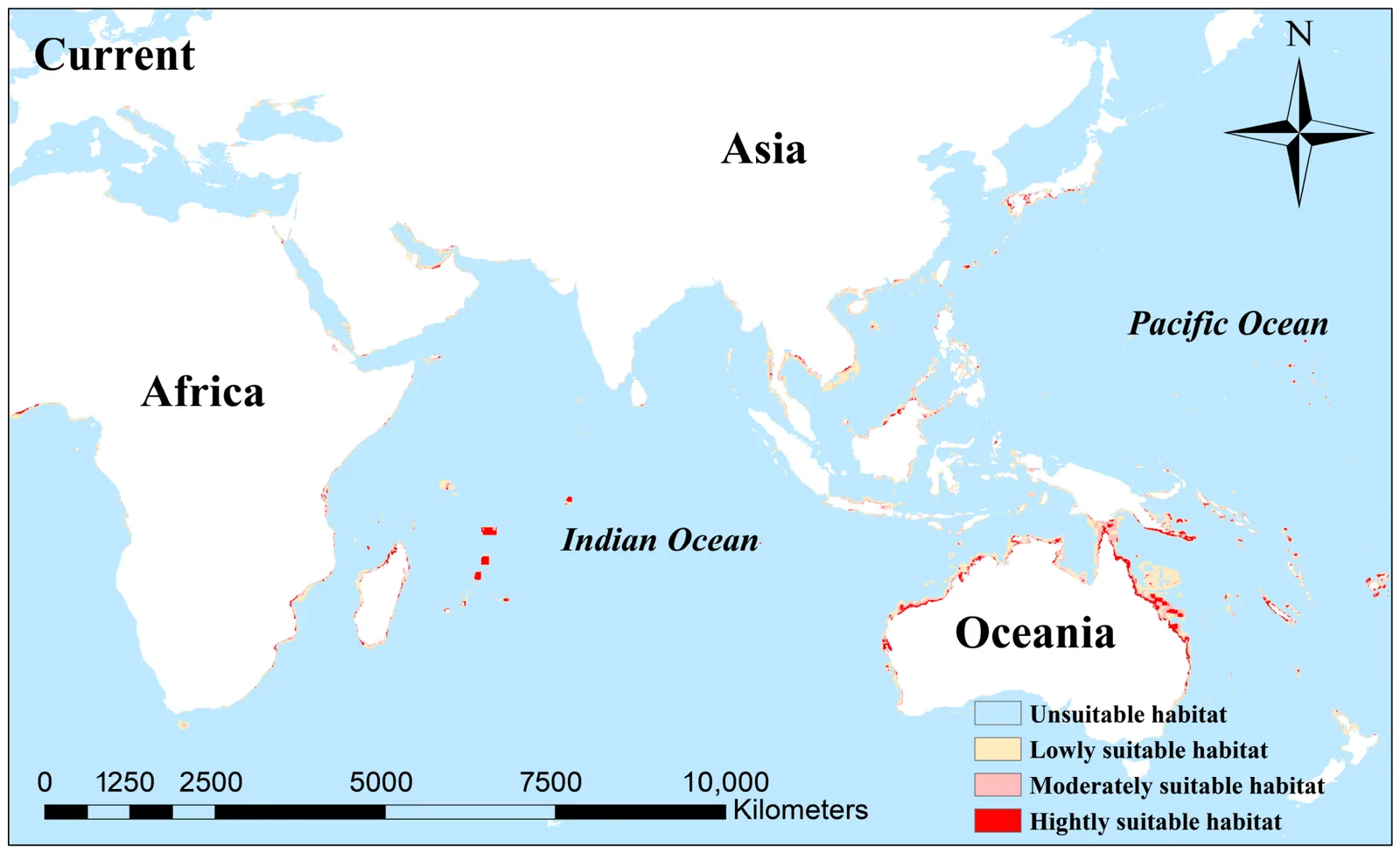
Global suitable habitat distribution of *T. peltata* under current environmental conditions simulated by the MaxEnt model.

The MaxEnt model was employed to simulate the global suitable habitats of *T. peltata* under current environmental conditions. Its overall suitable habitats are concentrated in tropical coral reef waters of the Indo-West Pacific Ocean (Figure 5). The red-colored highly suitable habitats are sporadically distributed along the coasts of Southeast Asian archipelagos, northern Australia, reefs in the northwestern Indian Ocean, and coastal reefs of the South China Sea in China, which serve as core areas of high predicted habitat suitability of this species. The light pink moderately suitable habitats extend zonally around the high-suitability core regions, covering the extensive coral reef belts of Southeast Asia, inshore waters of northern Australia and the continental shelf of the South China Sea, where coral population development is inferior to that in the core zones. The pale yellow low-suitability habitats only scatter in open peripheral waters adjacent to suitable areas and cannot support marginal habitats with low predicted suitability. The remaining light-blue areas, including most open oceans, high-latitude seas and offshore waters along the western coast of Africa, are classified as unsuitable habitats. The simulated distribution pattern is consistent with the natural biogeographic distribution of this species; Southeast Asia and northern Australia constitute its global distribution center, while the South China Sea of China lies at the northern marginal zone of its suitable distribution range.

### 3.4. Historical Habitat Distribution and Refugia Locations of T. peltata

**Figure 6:**
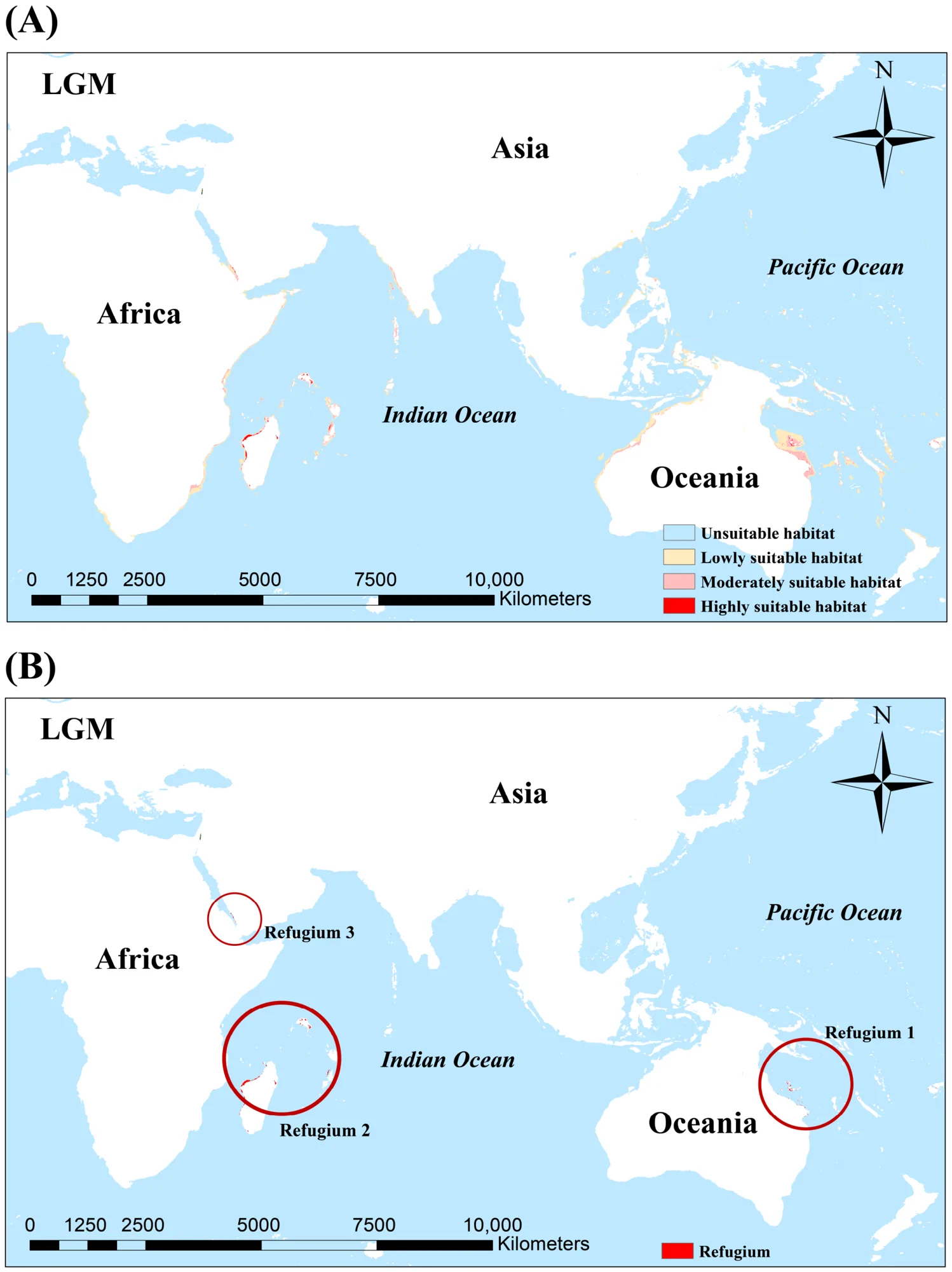
Distribution of potential suitable habitats and glacial refugia of *T. peltata* during the Last Glacial Maximum in the Indo-Pacific Ocean. (**A**) shows the graded spatial pattern of habitat suitability; (**B**) delineates three key glacial refugia (Refugium 1–3).

Affected by global cooling and substantial sea-level decline during the Last Glacial Maximum, extensive continental shelf exposure reshaped land–sea configurations, leading to dramatic shrinkage and severe fragmentation of suitable habitats for *T. peltata* compared with the present. Figure 6A reveals that scattered highly suitable habitats (dark red patches) are confined to the northeastern Australian coast, coastal Madagascar and the Red Sea-Gulf of Aden. Most areas, including Southeast Asia, the South China Sea and the central Indian Ocean, are entirely unsuitable, as the exposed Sunda Shelf eliminated most shallow reef substrates. Moderate and low-suitability habitats only sparsely adjoin high-quality zones and cannot sustain self-sustaining coral populations.

Three glacial refugia are identified in Figure 6B. Northeastern Australia serves as the primary refuge with well-connected habitats that supplied propagules for post-glacial recolonization (refugium 1). Coastal Madagascar maintained fragmented local populations as a secondary refuge in the southern Western Indian Ocean (refugium 2), while the semi-enclosed Red Sea–Gulf of Aden blocked cold oceanic intrusions and acted as an independent stable refuge in the northern Western Indian Ocean (refugium 3). Long-term geographic isolation of these refugia preserved separate coral populations through glacial disturbances and shaped the modern biogeographic pattern and genetic differentiation of this species across the Indo-West Pacific.

## 4. Discussion

### 4.1. Dominant Marine Environmental Drivers Shaping the Distribution of T. peltata

MaxEnt simulation results of this study indicated that water depth (EV6) was the most influential predictor in the fitted model, with a contribution rate of 44.76% and permutation importance up to 80.34%, which was highly consistent with the field ecological characteristics of this turbid-water coral. Field surveys of coral communities along the South China coast revealed that corals of the genus *Turbinaria* mostly inhabit continental shelf waters shallower than 20 m, and their populations almost disappear when water depth exceeds 75 m, matching the threshold obtained in this model perfectly [18]. Shallow coastal areas less than 3 m deep suffer severe wave disturbance coupled with extreme high temperatures in summer, which easily triggers large-scale coral bleaching. The depth range of 5–15 m, identified from the response curve (Figure 4) as the optimal habitat layer, balances the light demand of zooxanthellae and stable hydrodynamic conditions. When water depth exceeds 20 m, underwater light attenuates continuously, making it difficult for large coral colonies to develop. This vertical depth zonation rule accords with long-term field monitoring conclusions of coral reefs in Xuwen, Guangdong Province [19].

Distance to land was the second most important predictor with a contribution rate of 15.70%, fully reflecting the strong nearshore preference of *T. peltata*. Combined field measurements and model extraction data showed that the suitable offshore distance range for this coral was 0–45 km, as indicated by the response curve of the distance-to-land predictor, with optimal populations concentrated in turbid coastal waters 0–20 km from shore. Only scattered marginal individuals exist 20–45 km offshore, and predicted suitability becomes negligible beyond 45 km from land [4]. Unlike light-sensitive clear-water corals such as Acropora, corals of the genus *Turbinaria* have evolved high sediment tolerance. They can supplement energy via heterotrophic filter-feeding of terrigenous organic matter to compensate for insufficient photosynthesis under weak light, a unique physiological adaptation that determines their nearshore distribution pattern [20]. Sea surface temperature from May to October was the third key environmental factor with a contribution rate of 10.29%. Warm-season water temperature directly regulates coral calcification and reproduction cycles, and global coral niche simulations have verified that summer sea temperature acts as a core screening variable for tropical corals [21]. Nitrate concentration and seawater pH moderately affect coral tissue growth and skeletal calcification, while the remaining environmental variables each contribute less than 5% and only exert secondary regulatory effects. Jack-knife tests further confirmed that water depth contains unique predictive information unavailable from other variables, making it the core topographic factor shaping the spatial pattern of this coral across the entire basin scale.

### 4.2. Contemporary Biogeographic Pattern of T. peltata Across the Indo-West Pacific

Contemporary habitat simulations showed that areas of high predicted habitat suitability for *T. peltata* are restricted to tropical coral reef waters of the Indo-West Pacific Ocean. Southeast Asia and northern Australia constitute the global distribution center of this species, while the coastal waters of the South China Sea lie at the northern margin of its suitable range [5]. Dark red highly suitable habitats are scattered along the coasts of northern Australia, Southeast Asian archipelagos, the Red Sea and coastal reefs of the South China Sea. Light pink moderately suitable habitats extend zonally around core regions, covering the continental shelf of the South China Sea and extensive inshore waters of northern Australia. Pale yellow low-suitability patches only sporadically occur at the outer edges of reef systems and represent marginal habitats with low predicted suitability. Most open oceans, high-latitude seas and offshore waters off West Africa are classified as unsuitable habitats. The semi-enclosed basin topography of the Red Sea isolates cold, oligotrophic open-ocean water masses and forms continuous isolated high-suitability zones, a geographic isolation phenomenon validated by coral molecular phylogeny and paleoecological research [8].

The overall spatial distribution is fully consistent with global museum specimens and records from the GBIF and OBIS databases [22]. As a typical nearshore turbid-water coral, *T. peltata* rarely colonizes oligotrophic open-ocean reefs. Low winter sea surface temperatures in the South China Sea inhibit its further northward expansion, consistent with field survey results from Xisha and Sanya coral reefs [23]. Moderate water turbidity can alleviate stress from intense light and high temperature, rendering nearshore turbid zones core habitats for this species, which provides support for the ecological value of turbid-water corals [5]. Existing coral biogeographic research mainly focuses on branching Acropora corals adapted to clear water. This study supplements basin-wide distribution data of nearshore stress-tolerant *Turbinaria* corals and improves the theoretical system of Indo-West Pacific coral fauna evolution.

### 4.3. Habitat Contraction and Three Independent Glacial Refugia During the Last Glacial Maximum

Global cooling and a 120–135 m drop in sea level during the Last Glacial Maximum (LGM) exposed vast areas of continental shelf and reshaped land–sea configurations. The exposure of the Sunda Shelf eliminated most shallow reef substrates in Southeast Asia and the South China Sea, leading to severe contraction and fragmentation of suitable habitats for *T. peltata* [24]. As shown in Figure 6A, dark red highly suitable habitats are discretely distributed along three coastal zones: northeastern Australia, waters surrounding Madagascar, and the Red Sea–Gulf of Aden, while most regions of Southeast Asia and the central Indian Ocean are unsuitable. Three long-term candidate glacial refugia were delineated based on the contiguity of LGM areas with predicted suitability ≥ 0.8 (Figure 6B); because the LGM reconstruction relied on the present-day bathymetric grid combined with the reconstructed sea-level lowering rather than a fully dynamic palaeobathymetry, the modeled contraction of suitable habitat should be interpreted as a first-order approximation.

Refugium 1 (coastal northeastern Australia) represents the largest and best-connected glacial refuge across the whole basin. The steep eastern continental shelf retained continuous shallow reef slopes under low sea levels, with stable year-round water temperatures predicted to support extensive coral habitat. It may have served as a major source of propagules for coral dispersal toward Southeast Asia and the South China Sea after glaciation, and Quaternary reef sediment records of the Great Barrier Reef corroborate the long-term suitability of this region [6]. Refugium 2 (waters surrounding Madagascar) acts as a secondary refuge in the Southern Indian Ocean. Although scattered high-suitability patches persist on steep island fringes, the broad surrounding continental shelves were extensively exposed during the LGM, creating strong geographic isolation between patches that may have blocked gene flow over long periods and driven population genetic differentiation [25]. Refugium 3 (the Red Sea–Gulf of Aden) is the sole candidate refuge in the northern Western Indian Ocean. Its semi-enclosed geomorphology blocks cold-water intrusions and preserves unique local coral evolutionary lineages [8].

Most existing research on coral glacial refugia centers on clear-water Acropora species. This study reconstructs paleohabitats of turbid-water *T. peltata* and verifies that nearshore stress-tolerant corals also survived glacial climate fluctuations by relying on isolated low-latitude steep coastal zones, enriching the paleobiogeographic evolutionary system of hermatypic corals [25].

### 4.4. Conservation Implications for T. peltata Under Climate Change

Although *T. peltata* exhibits superior tolerance to high temperature and sedimentation compared with most sensitive hermatypic corals, its distribution is strictly constrained by water depth and offshore distance, making it vulnerable to combined threats from coastal engineering, offshore pollution and global warming [4]. The dark red core high-suitability zones identified in contemporary simulations (the Great Barrier Reef, archipelagic reefs of Southeast Asia, and coastal coral belts of the South China Sea) require priority protection and management. Submarine dredging and terrigenous nutrient input directly alter nearshore depth and turbidity regimes, completely destroying the optimal niche of this coral [23].

The three LGM glacial refugia identified in this study have maintained stable topographic and hydrological conditions for tens of thousands of years, representing natural climate-resilient hotspots. They are highly likely to become novel coral refugia under continuous future warming and should be prioritized for inclusion in transnational marine conservation networks [26]. As the northern marginal zone of this species, the South China Sea faces dual pressures of low temperature and human disturbance, requiring synchronous coastal pollution control and artificial coral reef restoration [23]. Current coral conservation planning generally ignores the buffering function of nearshore turbid reefs. This study quantitatively classifies basin-wide suitable habitats and identifies paleoclimatically stable refugia, providing spatial baselines and theoretical foundations for targeted protection of nearshore stress-tolerant corals [27].

## 5. Conclusions

This study established a robust MaxEnt niche model incorporating 21 non-collinear marine variables to systematically disentangle multi-scale environmental constraints governing the turbid-water coral *T. peltata* across the entire Indo-West Pacific. Bathymetry acted as the most influential predictor in the fitted model, shaping its spatial range, while nearshore proximity and warm-season seawater temperature constitute secondary limiting factors. Modern biogeographic zoning demonstrates that Southeast Asia and northern Australia represent the core evolutionary hotspot for *T. peltata*, whereas the South China Sea functions as its critical northern biogeographic margin sensitive to thermal fluctuations. Paleoclimatic reconstruction for the Last Glacial Maximum reveals severe range shrinkage driven by exposed continental shelves, and three geographically isolated candidate refugia are identified, which may have contributed to the species’ present-day distributional divergence; direct genetic or demographic evidence would be required to confirm their function as long-term population reservoirs. Beyond baseline habitat mapping, this work highlights the unique conservation value of turbid inshore reefs as climate buffering ecosystems. The identified glacial climate refugia serve as priority conservation targets under ongoing ocean warming, and the quantitative niche thresholds provided herein deliver targeted spatial guidance for differentiated regional coral reef protection strategies tailored to nearshore stress-tolerant coral taxa.

## Figures and Tables

**Table 1:** Global marine environmental variable data (including physical variables, chemical variables, and nutrient variables).

Environment Variable		Grid Layer Code
***Physical***	Depth	gb_depth
	Slope	gb_slope
	Aspect (East–West)	msp_asp_ew
	Aspect (North–South)	msp_asp_ns
	Distance to Land	gb_land_distance
	Distance to Port	port_distance
	Ice Concentration Annual	aq_ice_amn
	Ice Concentration (May–Oct)	aq_ice_sum
	Ice Concentration (Nov–Apr)	aq_ice_win
	Tide Average	kg_tide_average
	Wave Height	aq_waveheight
	Wind Speed	kg_wind_speed
	Surface Current	ecco2_uv_surface_current
	Euphotic Layer Bottom Depth	gc_zeu_mean
	Diffuse attenuation coefficient	bo_da_mean
	Maximum Temperature	bo_sst_maximum
	Average Temperature	bo_sst_average
	Minimum Temperature	bo_sst_minimum
	Range Temperature	bo_sst_range
	Sea Surface Temperature (May–Oct)	na_may_oct_sst
	Sea Surface Temperature (Nov–Apr)	na_nov_apr_sst
	Seabed Temperature	kg_b_temp
	Water Column Temperature	na_b_temp
	Salinity Mean	bo_salinity
	Bottom Salinity	na_b_salinity
	Photosynthetically Active Radiation	bo_parmean
***Chemical***	Average Chlorophyll-a	bo_chla_average
	Maximum Chlorophyll-a	bo_chla_maximum
	Minimum Chlorophyll-a	bo_chla_minimum
	Chlorophyll-a Range	bo_chla_range
	Summer (May–Oct) Maximum Chlorophyll-a	kg_chla_summax
	Winter (Nov–Apr) Maximum Chlorophyll-a	kg_chla_winmax
	Primary Productivity	aq_primprod
	pH	bo_ph
	Total Suspended Matter	gc_tsm_mean
***Nutrients***	Calcite	bo_calcite
	Nitrate	bo_nitrate
	Bottom Nitrate	kg_b_nitrate
	Phosphate	kg_phosphate
	Bottom Phosphate	kg_b_phosphate
	Silicate	bo_silicate
	Bottom Silicate	kg_b_silicate
	Dissolved Oxygen	bo_o2dis
	Bottom Dissolved Oxygen	kg_b_o2dissolve
	Saturated Oxygen	kg_s_o2saturate
	Bottom Utilized Oxygen	kg_b_o2utilized
	Particulate Organic Carbon	gc_poc_mean
	Particulate Inorganic Carbon	gc_pic_mean

## Data Availability

The raw data supporting the conclusions of this article will be made available by the authors on request.

## Supplementary Materials

The following supporting information can be downloaded at https://www.mdpi.com/article/10.3390/biology15181626/s1. Table S1: Occurrence records used for potential distribution modeling of *Turbinaria peltata*.

## Abbreviations

The following abbreviations are used in this manuscript:

AUC	Area Under the ROC Curve
GBIF	Global Biodiversity Information Facility
GMED	Global Marine Environment Datasets
LGM	Last Glacial Maximum
MaxEnt	Maximum Entropy Algorithm
OBIS	Ocean Biodiversity Information System
ROC	Receiver Operating Characteristic Curve
SDMs	Species Distribution Models
VIF	Variance Inflation Factor
